# Clinical significance of soluble programmed cell death‐ligand 1 in hepatitis B virus‐related hepatocellular carcinoma

**DOI:** 10.1002/mco2.225

**Published:** 2023-05-02

**Authors:** Zhongxia Yang, Xiaojun Liu, Ping Zhou, Yongwu Mao, Junfeng Li, Xiaorong Mao

**Affiliations:** ^1^ The First Clinical College of Lanzhou University Lanzhou Gansu China; ^2^ Department of Infectious Diseases The First Hospital of Lanzhou University Lanzhou Gansu China; ^3^ Second Division of Radiotherapy Gansu Provincial Hospital Lanzhou Gansu China

Dear Editor,

Primary liver cancer is the sixth most commonly diagnosed cancer and the third leading cause of cancer death worldwide in 2020,^1^ with hepatocellular carcinoma (HCC) comprising 75%–85% of cases.^1^ Hepatitis B virus (HBV) is an important etiological and disease progression factor in HCC. Nucleoside or nucleotide analogs control HBV virus replication, but not completely clear covalently closed circular DNA. According to the prediction of the International Agency for Research on Cancer (IARC) of the World Health Organization (WHO), the number of new cases and deaths due to liver cancer will further increase by 2040. Timely diagnosis and disease monitoring are very important to improve the prognosis of HCC. Tumors rely on different mechanisms to promote immune escape. Programmed cell death 1 and its ligand (PD‐1/PD‐L1) are important negative immune co‐stimulatory molecules. PD‐L1 can exist in different forms including membrane‐bound PD‐L1 (mPD‐L1), soluble PD‐L1 (sPD‐L1), nuclei PD‐L1, and exosome‐related PD‐L1. The multiple forms of PD‐L1 make the PD‐1/PD‐L1 signal pathway more complex. Among them, sPD‐L1 has attracted extensive attention because of its simple sampling, repeated detection, and lower cost. In this study, we investigated the plasma level and clinical significance of sPD‐L1 in HBV‐related HCC (HBV‐HCC).

The study included HBV‐HCC patients, HBV‐related cirrhosis (HBV‐LC) patients, and healthy controls (HC). All subjects were selected from the First Hospital of Lanzhou University (Lanzhou, China) from April 2021 to March 2022. There were early and advanced patients in HBV‐HCC group, whose average age was 55 years (53.933 ± 8.554) and 76 males (81.7%). The average age and sex of the HBV‐LC and HC were 49 years (49.792 ± 8.554) and 39 males (75%), 39 years (39.647 ± 10.006) and 40 males (80%), respectively. Results of biochemical (ALT, AST, TBIL, ALB), blood cells (leukocytes and platelets), and virological indexes (HBsAg and HBV‐DNA) between groups were statistically analyzed (Table S1).

The level of sPD‐L1 in peripheral blood of other different malignancy patients was increased. We noted that previous studies have focused on the predictive value of sPD‐L1 in HCC prognosis and changes before and after treatment, such as transcatheter arterial chemoembolization (TACE) and sorafenib. There was a lack of reports on the monitoring and diagnosis of sPD‐L1 in HBV‐HCC. In addition, in different studies, the level of sPD‐L1 in HCC is still inconsistent. Finkelmeier et al.^2^ showed that sPD‐L1 levels in HCC patients (median 0.5 ng/mL) were significantly lower than sPD‐L1 in the healthy individuals (0.78 ng/mL; *p* < 0.01) and the major etiologies of HCC included HBV infection. Another study reported that median sPD‐L1 concentration in patients with HBV‐HCC was 5.129 (range 0.140–12.391) ng/mL and in healthy controls was 0.836 (range 0.105–2.168) ng/mL (*p* < 0.001).^3^ We conducted further research on this basis. Our study showed that its level was 137.124 pg/mL (58.62–331.481 pg/mL), significantly higher than the level in HBV‐LC (median 76.163 pg/mL, range 40.166–152.828 pg/mL, *p* = 0.013) and in HC (median 55.696 pg/mL, range 30.942–73.226 pg/mL, *p* = 0.000) (Figure 1A). This difference between the studies may be attributed to the etiology of the included patients and whether they received treatment before grouping. Treatment for tumors often changes the tumor microenvironment, and antitumor therapy can reduce the level of sPD‐L1.^4^

**FIGURE 1:**
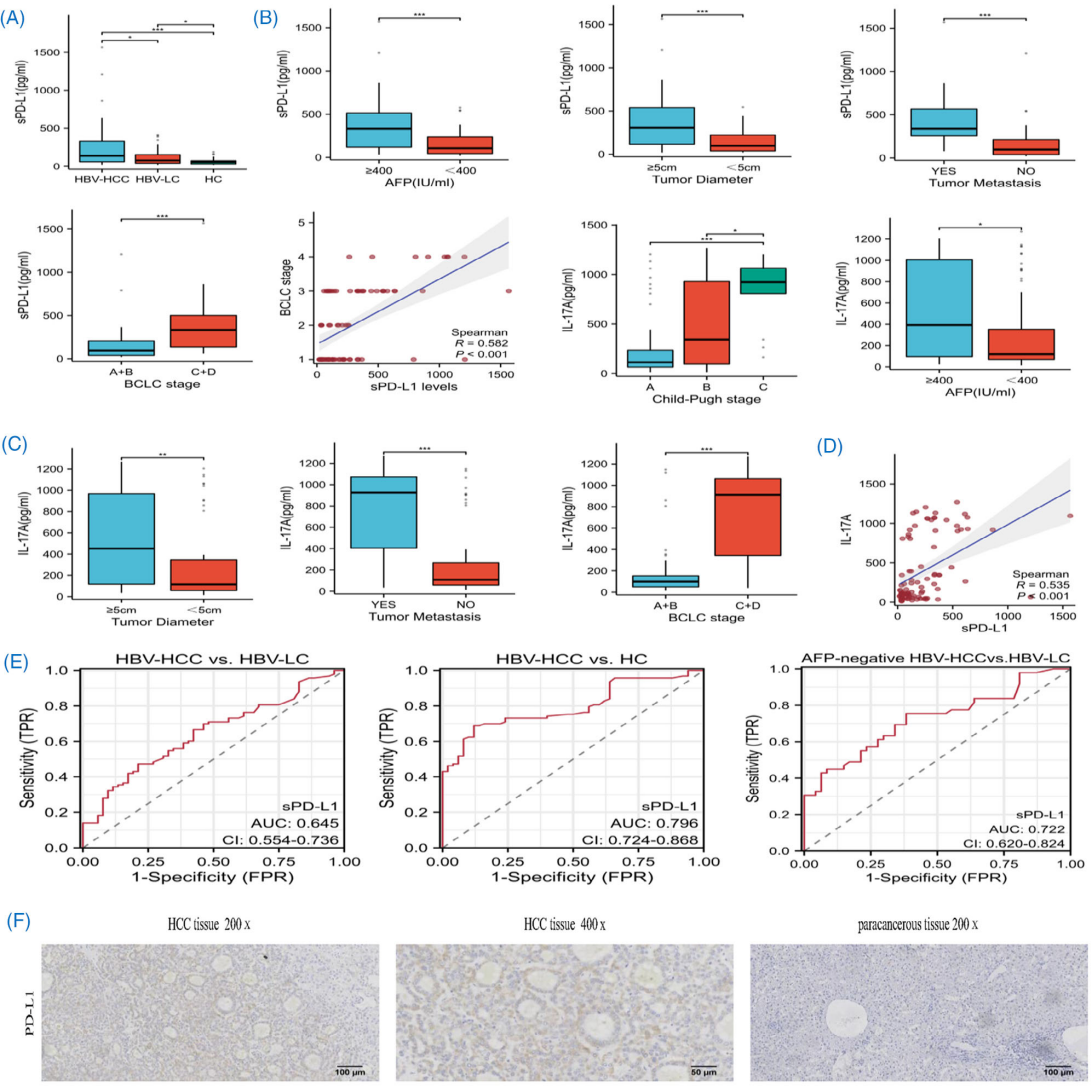
(A) Boxplots of sPD‐L1 concentrations in three groups. Patients with HBV‐HCC (*n* = 93) showed significantly higher plasma sPD‐L1 concentrations than HBV‐LC (*n* = 52) or HC (*n* = 50). (B) Comparison of plasma sPD‐L1 concentration in different subgroups of patients with HBV‐HCC. Statistical differences were shown in four subgroups, including AFP (*n* = 31 [≥400 IU/mL] vs. 62 [<400 IU/mL]), tumor diameter (*n* = 35 [≥5 cm] vs. 58 [<5 cm]), tumor metastasis (*n* = 26 [yes] vs. 67 [no]), and BCLC stage (*n* = 57 (A+B) vs. 36 [C+D]). Plasma sPD‐L1 concentration positively correlated with BCLC stage. (C) Comparison of plasma IL‐17A in different subgroups of patients with HBV‐HCC. SPD‐L1 level in Child–Pugh C (*n* = 55) was significantly higher than that in Child–Pugh B (*n* = 25) and A (*n* = 13). The higher level of plasma IL‐17A was found in patients with AFP ≥ 400 IU/mL (*n* = 31) than patients with AFP < 400 IU/mL (*n* = 62) (*p* = 0.046). Differences in level of plasma IL‐17A between patients with tumor diameter ≥5 cm (*n* = 35) and patients with tumor diameter <5 cm (*n* = 58) (*p* = 0.002). Significant differences in plasma level of sPD‐L1 whether there was metastasis (*p* < 0.001) (*n* = 26 (yes) vs. 67 [no]). Patients with BCLC stage C or D (*n* = 36) also had higher IL‐17A level than stage A or B (*n* = 57) (*p* < 0.001). (D) Plasma sPD‐L1 concentration positively correlated with plasma IL‐17A. (E) Receiver operator characteristic (ROC) curve of sPD‐L1 concentrations for patients with HBV‐HCC and patients with HBV‐LC and HC. The ability of sPD‐L1 diagnosed AFP‐negative HCC from HBV‐LC. (F) Immunohistochemistry staining results of PD‐L1 in intratumoral and adjacent liver tissues. Statistical analysis was performed using the Mann–Whitney *U* test and Kruskal–Wallis test. *p* < 0.05 was considered significant (*p < 0.05, ***p < 0.001). The correlation was analyzed using Spearman's *r* test. sPD‐L1, soluble programmed cell death ligand‐1; HBV‐HCC, hepatitis B virus‐related hepatocellular carcinoma; HBV‐LC, hepatitis B virus‐related liver cirrhosis; HC, healthy control, BCLC, Barcelona clinic liver cancer; IL‐17A, interleukin‐17A; *n* represents the included number of cases.

In order to clarify the expression of sPD‐L1 under different clinical characteristics, subgroup analysis was carried out in this study. SPD‐L1 level was higher in patients with alfa‐fetoprotein (AFP) greater than or equal to 400 IU/mL (median 331.946 pg/mL, range 117.591–513.904 pg/mL) than in patients with AFP less than 400 IU/mL (median 105.603 pg/mL, range 43.624–236.7 pg/mL, *p* = 0.000; Figure 1B). The level of sPD‐L1 in patients with tumor diameter greater than or equal to 5 cm (median 306.325 pg/mL, range 117.591–539.008 pg/mL) was noticeably higher than that in patients with tumor diameter less than 5 cm (median 98.233 pg/mL, range 38.124–221.432 pg/mL, *p* = 0.000; Figure1B). SPD‐L1 level was higher in patients with portal vein invasion or lung metastasis (median 337.993 pg/mL, range 254.989–566.008 pg/mL) than in patients without these conditions (median 97.054 pg/mL, range 38.77–211.197 pg/mL, *p* = 0.000; Figure1B). Patients with Barcelona clinic liver cancer(BCLC) A or B (median 97.054 pg/mL, range 37.909–207.246 pg/mL) had significant lower sPD‐L1 level than patients in stage C or D (median 333.549 pg/mL, range 135.61–501.334 pg/mL, *p* = 0.000; Figure 1B). Plasma sPD‐L1 concentration positively correlated with BCLC stage (Spearman *r* = 0.582, *p* < 0.001; Figure 1B). The results suggested that sPD‐L1 might be generated from liver tumor tissue and related to tumor growth and metastasis in HBV‐HCC. Similar results were obtained in other solid tumors, such as malignant melanoma and renal cell carcinoma. SPD‐L1 detection might be clinically useful for predicting tumor progression.

Some cytokines are involved in regulating the expression of PD‐L1 in tumor cells. Interleukin‐17A (IL‐17A), a pro‐inflammatory cytokine, is known to be associated with cancer. Inhibition of IL‐17A signaling may be a promising approach for hepatoma treatment. But only one study has reported that IL‐17A secreted from lymphatic endothelial cells promotes tumorigenesis by upregulation of PD‐L1 in hepatoma stem cells in vitro so far.^5^ We wanted to further observe the expression of IL‐17A in HBV‐HCC and its relationship with sPD‐L1 in patients. We compared the IL‐17A level in different subgroups. Similarly, significant difference was seen in five subgroups, including Child–Pugh, AFP, tumor diameter, tumor metastasis, BCLC stage (Figure 1C). The results also showed that IL‐17A might be associated with tumor load and tumor invasiveness of HBV‐HCC. Both sPD‐L1 and IL‐17A had no significant difference in subgroup analysis based on age, gender, HBV‐DNA, model for end‐stage liver disease score (MELD), albumin‐bilirubin grade (ALBI), degree of tumor differentiation and microvascular invasion (MVI) (Table S2).

IL‐17A promotes immune escape of hepatoma stem cells partly through upregulation of PD‐L1 expression.^5^ SPD‐L1 is a form of PD‐L1. We hypothesized that IL‐17A may also affect the expression level of sPD‐L1. Then we analyzed the correlation between two. Results demonstrated that the plasma level of IL‐17A positively correlated with the expression level of sPD‐L1 in HBV‐HCC (*r* = 0.533, *p* < 0.001, Figure 1D). IL‐17A may be involved in the regulation of PD‐1/PD‐L1 signaling pathway in HBV‐HCC microenvironment. But more research is required to understand the role and the mechanism of it.

SPD‐L1 can be used as a blood marker for the diagnosis of some diseases. But little is known about the diagnostic ability of sPD‐L1 in HBV‐HCC. In the study, receiver operator characteristic (ROC) curve showed that the area under the ROC curve (AUC), sensitivity, and specificity of sPD‐L1 in distinguishing HBV‐HCC from HBV‐LC were 0.645, 47.3%,78.8%, respectively (Figure 1E), and the cutoff vale was 156.38 pg/mL. The AUC, sensitivity, and specificity of sPD‐L1 in distinguishing HBV‐HCC from HC were 0.796, 68.8%, 88%, respectively (Figure 1E). AFP‐negative HCC is not easily detected. Furthermore, we evaluated the diagnosis potential for distinguishing AFP‐negative HCC from cirrhosis, with AUC of 0.722, sensitivity of 75.5%, specificity of 61.7%. The results indicated that sPD‐L1 has the superior diagnosis potential for HBV‐HCC from cirrhosis or healthy person.

This study also included 31 HCC patients who received surgical resection. The expression of PD‐L1 in liver tumor tissues was higher than in matched paracancerous tissues (Figure 1F). PD‐L1 expression was positive in 14 (48.39%) of 31 surgically resected cancer tissues. Three patients had positive PD‐L1 expression in adjacent liver tissues. The study also showed that plasma level of sPD‐L1 was uncorrelated to the expression of PD‐L1 on tumor cells. This may be due to other sources of sPD‐L1 besides hydrolysis of mPD‐L1. And in tumor microenvironment, both tumor cells and immune cells could produce sPD‐L1.

In conclusion, we elucidated that plasma level of sPD‐L1 was increased in HBV‐HCC patients and significantly related to malignant biological behavior of HBV‐HCC. SPD‐L1 also had a value to help diagnose HBV‐HCC. These suggested that sPD‐L1 might be a potential biomarker for tumor monitoring and diagnosis in HBV‐HCC. Besides, inflammatory factor IL‐17A may affect the production of sPD‐L1.

## AUTHOR CONTRIBUTIONS

Research design: Zhongxia Yang and Xiaojun Liu. Experiment performed: Zhongxia Yang, Ping zhou, and Junfeng Li. Data collection: Zhongxia Yang, Ping zhou, and Yongwu Mao. Data analyses and manuscript draft: Zhongxia Yang and Xiaojun Liu. Critical revision of the article: Xiaorong Mao. All authors have read and approved the final version of the manuscript.

## CONFLICT OF INTEREST STATEMENT

The authors declare no conflicts of interest.

## ETHICS STATEMENT

This study was approved by Ethics Committee of the First Hospital of Lanzhou University (LDYYLL2021‐319). Written informed consent was obtained from all participants.

## Supporting information

Supporting Information

## Data Availability

The data supporting this study can be obtained from the corresponding author.
