# Supplementary material for: Clinical significance of soluble programmed cell death‐ligand 1 in hepatitis B virus‐related hepatocellular carcinoma
Source: MedComm (2020). 2023 May 2;4(3):e225. doi: 10.1002/mco2.225 (PMC10154365; doi:10.1002/mco2.225)
Supplement: Supplementary file 1 — Supporting Information [file MCO2-4-e225-s001.pdf]

Clinical Significance of soluble Programmed Cell Death-ligand 1 in Hepatitis B Virus Related Hepatocellular Carcinoma

Zhongxia Yang[1,2], Xiaojun Liu[3], Ping zhou[2], Yongwu Mao[2], Junfeng Li[2], Xiaorong Mao[1,2]

[1] The First Clinical College of Lanzhou University, Lanzhou, China
[2] Department of Infectious Diseases, The First Hospital of Lanzhou University, Lanzhou, Gansu, China
[3] Second Division of Radiotherapy, Gansu Provincial Hospital, Lanzhou, Gansu, China

Material and methods

Patients selection

The study included 93 HBV-related HCC(HBV-HCC), 52 HBV-related LC(HBV-LC), 50 healthy controls (HC). All subjects were collected in the First Hospital of Lanzhou University (Lanzhou, China) from April 2021 to March 2022. The diagnostic of HCC basis was the pathology or magnetic resonance imaging (MRI), computed tomography (CT)in concordance with the National Comprehensive Cancer Network (NCCN) guideline (2019). HBV-related HCC was HCC positive for hepatitis B surface antigen (HBsAg). All patients had never received any therapies before, including chemotherapy, radiotherapy, targeted therapy, immunotherapy or surgical resection. HBV- LC was diagnosed based on HBsAg, physical examination, biochemical, endoscopic, ultrasound and radiological signs. All patients (HCC and LC) had no serious infection, autoimmune disease, pregnancy, or other tumors. HC were collected from the physical examination. This study was approved by Ethics Committee of the First Hospital of Lanzhou University (LDYYLL2021-319).

Specimen collection

Morning fasting blood samples from HCC, LC and HC were collected in EDTA after enrollment. Plasma was obtained by centrifuging whole blood at 3000g for 20 minutes at 4°C, and plasma samples were then stored at −80°C until further processing.

A total of 31 HCC received resection after grouping, whose tumorous and adjacent nontumor liver tissues were collected during surgery. The collected tissue samples were made into paraffin blocks for use.

Quantitative detection of HBsAg and HBV DNA

Serum HBsAg was measured according to the procedure of the Roche Cobas E 601 chemiluminescence immune analyzer and HBV DNA level was assayed by the Polymerase Chain Reaction (PCR) (TIANLONG, Suzhou, China) with a detection limit of 100 IU/ml.

ELISA detection of plasma samples

Levels of sPD-L1, IL-17A in plasma were measured using commercial enzyme-linked immunosorbent assay (ELISA) kits (PD-L1(Proteintech, Chicago, USA). IL-17A (Elabscience Biotechnology, Wuhan, China). Experimental was conducted according to manufacturer's instructions. The standards, test samples were pipetted into the 96-wells and incubate at 37℃. Biotinylated detection antibodies were added to each well and incubate, then unbound biotinylated antibodies washed off by washing buffer. HRP-conjugated working solution was pipetted into each well and incubated, after that the wells were washed and substrate stop solution was added. The optical absorbance was measured at 450 nm using a microplate reader (Thermo Scientific Varioskan Flash, Massachusetts, USA). Protein levels were calculated using standard curves.

Immunohistochemistry

The paraffin-embedded tumor samples and adjacent liver tissues were sliced and then immunohistochemical staining to detect PD-L1. Briefly, after deparaffinization, rehydration, antigen retrieval, endogenous peroxidase inactivation and non-specific binding blockade, slides were incubated with primary antibodies (1:250, ab213524, Abcam, U.K) at 4°C overnight. Then, the slides were incubated with a corresponding secondary antibody (ZSGB-BIO, Beijing, China) for 15 min, followed by incubation with horseradish enzyme labeled streptomyces ovalbumin working solution. After that the slides were stained by diaminobenzidine (DAB) solution at room temperature for 4 min and re-stained with hematoxylin. The negative control was performed by omitting the primary antibodies.

The tissue sections were evaluated separately and blindly by two pathologists (Dr Liu and Dr Jin). Five fields in each specimen were selected randomly for analysis. PD-L1 staining was estimated using the Tumor Proportion Score (TPS). TPS was defined as the number of positive tumor cells divided by the total number of viable tumor cells multiplied by 100%. TPS≥1% was considered positive in the study, otherwise TPS<1% as negative.

Statistical analysis

All statistical analysis were conducted by IBM SPSS statistics 26.0 and R software 3.6.3.(R Foundation). All data are presented as absolute values, percentages, means ± SD, or medians and interquartile range. Independent -Samples T test, Mann–Whitney U test and Kruskal-Wallis Test was conducted for comparison between groups. The association between sPD-L1 and other factors was explored by Spearman's correlation. Cohen's kappa coefficient was used to assess the agreement between the evaluations of PD-L1 staining. ROC curves were generated to assess the diagnostic value of sPD-L1. For all analysis, a value of $p < 0.05$ was considered statistically significant.

Table S1 Demographic and clinicopathological features

Characteristics	HBV-HCC	HBV-LC	HC
Patients(n)	93	52	50
Age(years)($\bar{X}$±s)	53.933±8.554	49.792±8.554[a]	39.647±10.006[ab]
Gender (Male/Female)	76/18	39/13	40/10
ALT(U/L) (median, range＊)	45.75(7.0-553)	28.15(6.5-406)[a]	21.00(8.0-37)[ab]
AST(U/L) (median, range＊)	49(11.0-553)	42(12.5-244)	21(14.0-34)[ab]
TBIL ((µmol/L) (median, range＊)	24.50(8.5-962.20)	23.00(8.70-149.60)	16.36(6.46-40.98)[ab]
ALB(g/L) ($\bar{X}$±s)	38.614±7.366	37.968±6.554	42.186±3.363[ab]
WBC(×10^9/L) ($\bar{X}$±s)	3.777± 1.447	3.061± 1.209[a]	4.202±0.501[ab]
PLT(×10^9/L) ($\bar{X}$±s)	105.624± 58.430	71.731±39.551[a]	186.569± 46.898[ab]
HBsAg（IU/ML）			
≥250(n, (%))	53(56.38)	43(82.69)	-
< 250(n,(%))	41(43.62)	9(17.31)	-
HBV DNA（IU/L）			
< 100(n,%)	63(67.02)	38(73.08)	-
100-10^3(n, %)	13(13.83)	3(5.77)	-
> 10^3(n,%)	18(19.15)	11(21.15)	-

＊ The values in parenthesis are minimum and maximum; [a] Compared with HBV-HCC, p<0.05 [b] compared with HBV-LC, p<0.05

HBV- HCC, hepatitis B virus related hepatocellular carcinoma; HBV-LC, hepatitis B virus related liver cirrhosis; HC, healthy control.

Table S2 Comparison of sPD-L1, IL-17A and clinicopathological features in patients with HBV-HCC

Characteristics	n	sPD-L1(pg/ml) median (upper and lower quartile)	p	IL-17A(pg/ml) median (upper and lower quartile)	p
Age(years)					
＜60	64	172.913(87.046 - 333.549)	P=0.197	179.202(77.205 - 513.86)	P=0.914
≥60	29	110.248(38.77 - 291.474)		129.391(57.502 - 828.677)	
Gender					
Male	73	140.337(74.5 - 330.084)	P=0.597	232.41(76.742 - 902.688)	P=0.212
Female	20	123.882(42.887 - 333.34)		124.749(53.162 - 261.949)	
HBV DNA（IU/L）					
＜1000	70	128.492(47.783 - 270.671)	P=0.067	152.66(64.634 - 374.114)	P=0.065
≥1000	23	215.149(106.735 - 410.743)		393.407(111.068 - 939.262)	
Child-pugh					
A	55	103.657(43.836 - 310.779)	P=0.098	112.781(63.718 - 236.617)	P=0.000
B	23	176.599(88.03 - 377.011)		343.452(94.474 - 929.504)	
C	15	269.211 (113.756 - 342.437)		923.077(805.508- 1064.662)	
AFP (ng/mL)					
＜400	62	331.946(117.591 - 513.904)	P=0.000	118.033(69.525 - 352.741)	P=0.046
≥400	31	331.946(117.591 - 513.904)		392.621(95.428 - 1005.116)	
ALBI (grade)					
1	43	121.426(44.259 - 268.362)	P=0.259	120.106(53.145 - 266.569)	P=0.054
2	35	146.640(85.193 - 393.001)		264.163(78.287 - 968.727)	
3	15	253.752 (105.405-333.549)		451.877（188.543-915.523）	
BCLC stage					
A+B	57	97.054(37.909 - 207.246)	P=0.000	97.927(49.693 - 152.66)	P=0.000
C+D	36	333.549(135.61 - 501.334)		912.5(344.059 - 1061.501)	
Maximal tumor diameter（cm）					
＜5	58	98.233(38.124 - 221.432)	P=0.000	114.053(56.823 - 343.452)	P=0.002
≥5	35	306.325(117.591 - 539.008)		451.877(116.121 - 968.727)	
Tumor metastasis*					
YES	26	337.993(254.989 - 566.008)	P=0.000	926.29(405.746 - 1071.849)	P=0.000
NO	67	97.054(38.77 - 211.197)		107.707(57.049 - 266.569)	
Differentiation Degree					
High	3	95.685(58.969 - 168.446)	P=0.928	112.781(76.936 - 132.721)	P=0.837
Median	8	85.877(42.413 - 131.413)		69.702(45.897 - 92.222)	
Low	20	74.849（38.296-197.032）		83.262(55.020-115.882)	
MVI					
M0	16	99.671(42.887 - 227.689)	P=0.133	66.307(48.132 - 119.08)	P=0.654
M1+ M2	15	44.259(36.874 - 98.384)		89.781(63.516 - 112.153)	

* Tumor metastasis includes portal vein infiltration and lung metastasis

sPD-L1, soluble programmed cell death ligand-1; IL-17A, Interleukin 17A; HBV- HCC, hepatitis B virus related hepatocellular carcinoma; AFP, Alpha fetoprotein; BCLC, Barcelona Clinic Liver Cancer.
